# Interleukin-1 Receptor-Induced Nitric Oxide Production in the Pancreas Controls Hyperglycemia Caused by Scorpion Envenomation

**DOI:** 10.3390/toxins12030163

**Published:** 2020-03-05

**Authors:** Mouzarllem B. Reis, Jefferson Elias-Oliveira, Marcella R. Pastore, Simone G. Ramos, Luiz G. Gardinassi, Lúcia H. Faccioli

**Affiliations:** 1 Departamento de Análises Clínicas, Toxicológicas e Bromatológicas, Faculdade de Ciências Farmacêuticas de Ribeirão Preto, Universidade de São Paulo, São Paulo 14040-903, Brazil; mouzarllem@gmail.com (M.B.R.); jeffersonelias@usp.br (J.E.-O.); marcella.rpastore@gmail.com (M.R.P.); gustavogardinassi@usp.br (L.G.G.); 2 Departamento de Patologia e Medicina Legal, Faculdade de Medicina de Ribeirão Preto, São Paulo 14049-900, Brazil; sgramos@fmrp.usp.br

**Keywords:** scorpion venom, nitric oxide, hyperglycemia, inflammation, pancreas

## Abstract

*Tityus serrulatus* causes numerous scorpion envenomation accidents and deaths worldwide. The symptoms vary from local to systemic manifestations, culminating in pulmonary edema and cardiogenic shock. Among these events, transitory hyperglycemia is a severe manifestation that influences pulmonary edema, hemodynamic alterations, and cardiac disturbances. However, the molecular mechanism that leads to increased glucose levels after *T. serrulatus* envenomation remains unknown. This study aimed to investigate our hypothesis that hyperglycemia due to scorpion envenomation involves inflammatory signaling in the pancreas. The present study showed that *T. serrulatus* venom induces the production of IL-1α and IL-1β in the pancreas, which signal via IL-1R and provoke nitric oxide (NO) production as well as edema in β-cells in islets. *Il1r1*^−/−^ mice were protected from transitory hyperglycemia and did not present disturbances in insulin levels in the serum. These results suggest that the pathway driven by IL-1α/IL-1β-IL-1R-NO inhibits insulin release by β-cells, which increases systemic glucose concentration during severe scorpion envenomation. A supportive therapy that inhibits NO production, combined with antiserum, may help to prevent fatal outcomes of scorpion envenomation. Our findings provide novel insights into the design of supportive therapy with NO inhibitors combined with antiscorpion venom serum to overcome fatal outcomes of scorpion envenomation.

## 1. Introduction

Scorpion envenomation is a serious public health problem in tropical countries [1]. In Brazil, the number of accidents caused by these arthropods is higher than the number of accidents caused by all other venomous animals combined [2]. *Tityus serrulatus* (Ts) is the most prominent species in this scenario, because this scorpion is highly adapted to urban centers and reproduces by parthenogenesis [3]. Moreover, chemical control of scorpions is still a challenge.

Scorpion stings result in a wide range of symptoms, classified as minor, mild, and severe [1]. Most cases of scorpion envenomation culminate in minor manifestations, which include only local effects, such as intense pain, erythema, and redness [1,4]. Mild manifestations include tachycardia, vomiting, agitation, and sweating, among others. Severe manifestations comprises all minor and mild symptoms accompanied by pulmonary edema and cardiac manifestations [1,4]. Interestingly, envenomed patients often present transitory hyperglycemia that is directly correlated with envenomation severity [4]. Yugandhar et al. [5] demonstrated that insulin treatment in humans stung by the Indian red scorpion improved the clinical outcome and prevented pulmonary edema. In addition, de Oliveira et al. [6] suggested that diabetic victims of scorpionism can be considered a high-risk group, as nonobese diabetic mice were highly susceptible to envenomation by Ts venom (TsV). However, the molecular mechanisms that account for increased glucose levels in circulation remain unknown. 

Pattern recognition receptors (PRRs), such as Toll-like receptor 2 (TLR2), Toll-like receptor 4 (TLR4), cluster of differentiation 14 (CD14), and cluster of differentiation 36 (CD36), recognize TsV toxins and activate a range of molecular pathways in innate immune cells [7,8,9,10]. These interactions activate transcriptional factors such as activator protein 1 (AP-1), nucelar factor kappa-light-chain-enhancer of activated B cells (NF-κB), and peroxisome proliferator-activated receptor gamma (PPAR-γ), which induce the formation of lipid bodies, as well as the production of eicosanoids and cytokines. These inflammatory cascades are directly correlated to the severity of envenomation and caused death in experimental mouse models of envenomation [11,12]. Diabetes is caused by a range of immunological mechanisms [13]. Based on this evidence, our study hypothesized that hyperglycemia resulting from scorpion envenomation is caused by inflammatory signaling in the pancreas.

## 2. Results

### 2.1. IL-1 Receptor Regulates Scorpion Venom-Induced Hyperglycemia and Insulin Levels

**Figure 1:**
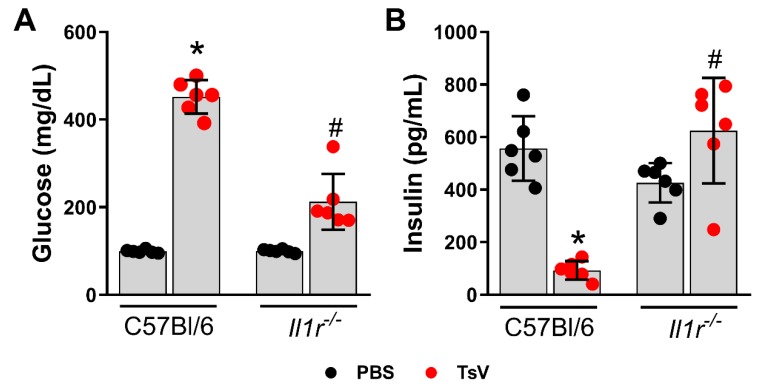
*Tityus serrulatus* venom (TsV) induces hyperglycemia and decreases insulin levels during severe scorpion envenomation. C57BL/6 and *Il1r1*^−/−^ mice were inoculated with phosphate-buffered saline (PBS) or with 180 µg.kg^−1^ of TsV and euthanized after 60 min to quantify circulating glucose using strip tests (**A**) and serum insulin by ELISA (**B**). Data are expressed as mean ± standard deviation (*n* = 6) and represent one out of two independent experiments. * *p* < 0.05 (PBS vs. TsV); ^#^ *p* < 0.05 (*Il1r1*^−/−^ TsV vs. C57BL/6 TsV).

Previous studies have demonstrated the importance of interleukin 1 beta/interleukin 1 receptor (IL-1β/IL-1R) signaling during envenomation by TsV [11,14,15]. This study investigated whether this signaling axis impacts insulin secretion and glycemia during scorpion envenomation in mice. C57BL/6 (WT) and *Il1r1*^−/−^ mice presented distinct insulin levels after TsV challenge. WT mice presented high levels of glucose in circulation 1 h after TsV inoculation, whereas it was significantly lower in *Il1r1*^−/−^ mice (Figure 1A). Consistent with this phenotype, WT mice presented reduced levels of insulin in serum, whereas *Il1r1*^−/−^ animals exhibited preserved insulin production in response to scorpion envenomation (Figure 1B). These results suggested that an IL-1R-dependent mechanism regulates insulin secretion and has a significant impact on glucose levels upon scorpion envenomation. 

### 2.2. Nitric Oxide (NO), Chemokines, and Myeloperoxidase (MPO) are Downregulated in Il1r1^−/−^ Animals

**Figure 2:**
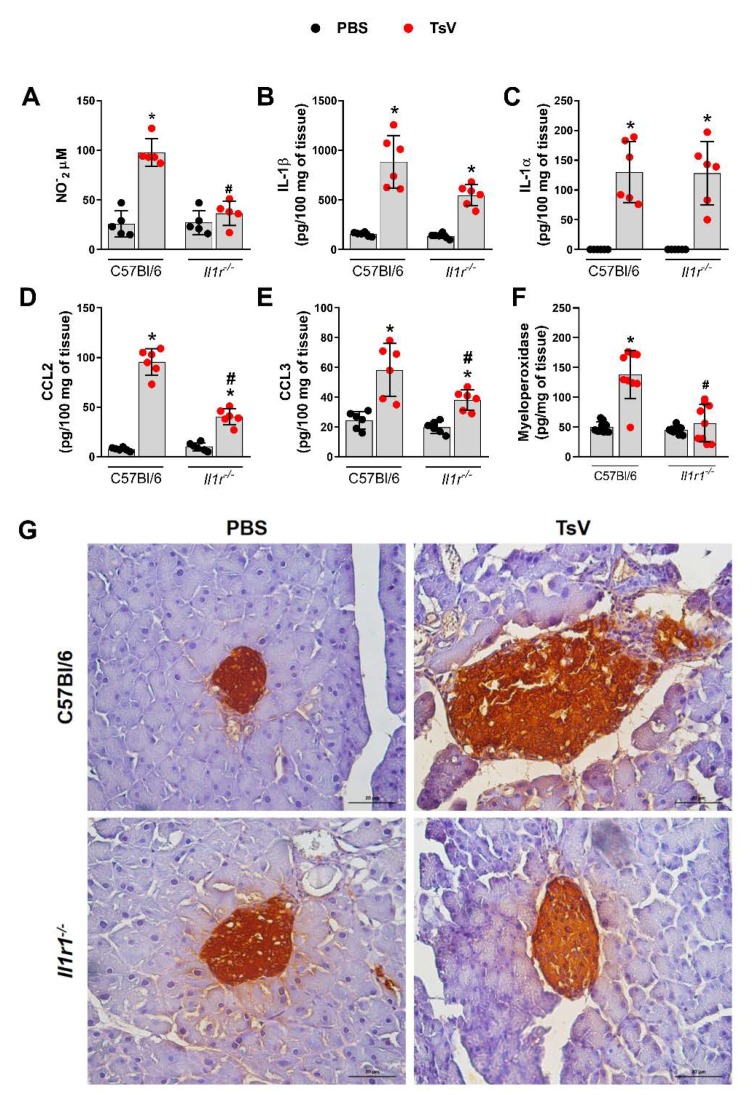
Scorpion envenomation induces pancreatic inflammation and edema in β-cell of islets. C57BL/6 and *Il1r1*^−/−^ mice were inoculated with phosphate-buffered saline (PBS) or with 180 µg.kg^-1^ of *Tityus serrulatus* venom (TsV) and euthanized after 60 min. The pancreas was removed and homogenized for the quantification of nitric oxide (NO) (**A**), IL-1β (**B**), IL-1α (**C**), CCL2 (**D**), CCL3 (**E**), and myeloperoxidase (**F**) or immunohistochemistry analysis (**G**) (scale bar, 20 µm). Data are expressed as mean ± standard deviation (*n* = 6) and represent one out of two independent experiments. * *p* < 0.05 (PBS vs. TsV); ^#^ *p* < 0.05 (*Il1r1*^−/−^ TsV vs. C57BL/6 TsV).

To investigate how the IL-1R pathway influences insulin production, several inflammatory mediators in the pancreas of lethally envenomed or phosphate-buffered saline (PBS)-inoculated mice were quantified 1 h after envenomation or PBS inoculation. The concentrations of NO, IL-1β, IL-1α, chemokine (C-C motif) ligand 2 (CCL2), chemokine (C-C motif) ligand 3 (CCL3), and MPO were increased in pancreatic homogenates from WT mice after envenomation (Figure 2A–F). Moreover, levels of the IL-1R signaling cytokines, IL-1β, and IL-1α, remained elevated in the pancreas of *Il1r1*^−/−^ mice compared with those in the pancreas of WT mice (Figure 2B,C). However, the concentrations of downstream effectors of IL-1R signaling, such as CCL2, CCL3, MPO, and NO, were significantly reduced in the pancreas of *Il1r1*^−/−^ mice (Figure 2A,D–F). Immunohistochemistry of pancreatic islets from envenomed WT mice demonstrated that β-cells exhibited increased size and were surrounded by a marked inflammatory infiltrate, which was not observed in the β-cells of *Il1r1*^−/−^ mice (Figure 2G). These results demonstrate that IL-1R signaling induces inflammation, production of mediators that may disturb the homeostasis of β-cells, and insulin secretion in response to high levels of circulating glucose after envenomation by TsV.

### 2.3. Inhibition of NO Production Prevents Scorpion Venom-Induced Hyperglycemia

**Figure 3:**
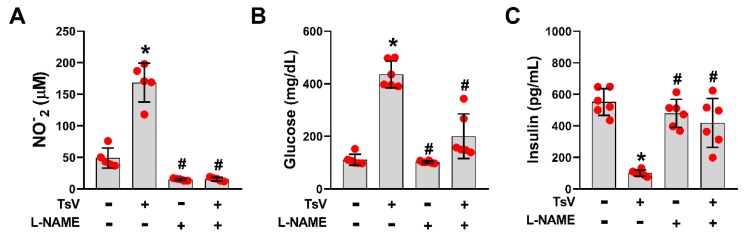
Inhibition of nitric oxide (NO) production reverses hyperglycemia during scorpion envenomation. C57BL/6 mice were treated with 100 mg.kg^−1^ of L-NAME via i.p. route 15 min prior to administering 180 µg.kg^−1^ of *Tityus serrulatus* venom (TsV). Phosphate-buffered saline (PBS)-inoculated mice and mice untreated with L-NAME and/or lethal dose of TsV were used for comparison. After 60 min, the pancreas was removed and homogenized for the quantification of NO (**A**), glucose (**B**), and insulin levels (**C**). Data are expressed as mean ± standard deviation (n = 6) and represent one out of two independent experiments. * *p* < 0.05 (PBS vs. TsV); ^#^ *p* < 0.05 (C57BL/6 L-NAME; C57BL/6 L-NAME + TsV vs. C57BL/6 TsV).

**Figure 4:**
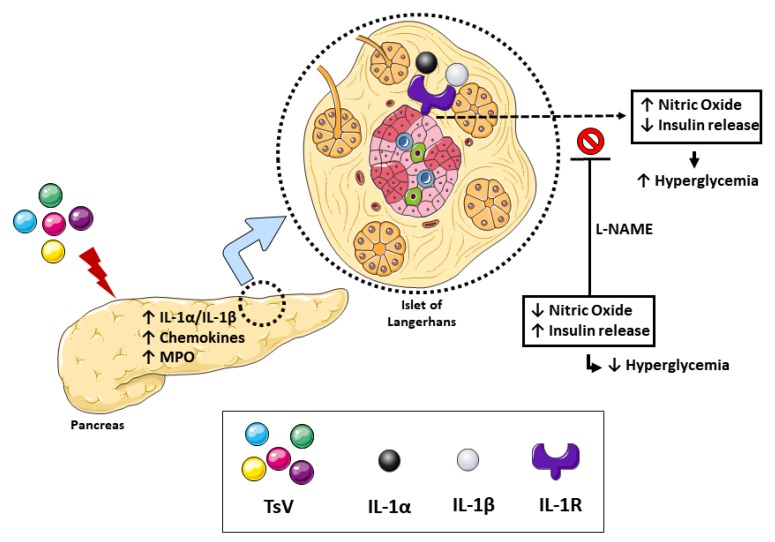
Tityus *serrulatus* venom (TsV) reaches the pancreas and induces the production of inflammatory mediators, including IL-1α/IL-1β, chemokines, and myeloperoxidase (MPO). Cytokines of the IL-1 family bind to IL-1R in the islets of Langerhans and induce the production of nitric oxide, thereby inhibiting the release of insulin and leading to hyperglycemia. The inhibition of nitric oxide production by L-NAME can increase circulating insulin levels and decrease TsV-induced transient hyperglycemia.

NO is an important mediator involved in the inhibition of insulin release [16]. Therefore, this study aimed to determine whether NO synthase (NOS) and NO production mediate the downstream effects of IL-1R signaling in scorpion venom-induced hyperglycemia. For this, WT mice were inoculated with a lethal dose of TsV and treated with the NOS inhibitor, N(G)-Nitro-L-arginine methyl ester (L-NAME). As expected, this treatment reduced NO levels in the pancreas (Figure 3A). Strikingly, the inhibition of NO production reduced both glucose and insulin levels in the circulation of envenomed mice (Figure 3B,C). Collectively, these results demonstrate that the IL-1/IL-1R/NOS/NO pathway promotes hyperglycemia during scorpion envenomation. The proposed mechanism is represented in Figure 4.

## 3. Discussion

TsV comprises compounds from different classes, such as hyaluronidases, metalloproteinases, serine proteases, high- and low-molecular weight proteins, and neurotoxins. These compounds are involved in the modulation of ion channels and the consequent autonomic modulation during envenomation [3]. This modulation leads to sympathetic hyperactivation with a marked increase in catecholamines, leading to symptoms such as tachycardia, hypertension, mydriasis, myocarditis, and cholinergic exacerbation, and other reflecting symptoms, such as bradycardia, hypotension, vomiting, bronchospasms, and priapism [1]. 

Hyperglycemia represents a clinical sign of severe scorpion envenomation [4]. High glucose levels are directly associated with the emergence of pulmonary edema and cardiac dysfunction [4]. However, the mechanisms that account for this process were not known. Scorpion venom from different species have been shown to induce acute pancreatitis in children, which correlates with vomiting and abdominal pain [17]. Moreover, levels of serum amylase and lipase, which are markers of pancreatic damage, are often elevated in patients stung by scorpions [18]. In addition, the increase in catecholamines during scorpion envenomation influences the deregulation of hormones such as glucagon and cortisol, as well as the inhibition of insulin release [19]. The increase in catecholamines, cortisol, and glucagon is opposed to the anabolic actions of insulin, leading to an inability of the organs to use glucose, thus contributing to the appearance of clinical symptoms and multisystem organ failure [20].

The inflammatory response has an important role in promoting the clinical manifestations observed during envenomation [12]. TsV toxins are recognized by PRRs and are able to induce a massive increase in cytokines and lipid mediators systemically in pulmonary and peritoneal macrophages [9]. This phenomenon is stimulated by crude venom and isolated toxins Ts1, Ts2, and Ts6 [7]. IL-1β is responsible for the severe manifestations of envenomation, such as pulmonary edema [11]. The antagonism or absence of the IL-1R receptor improved survival and clinical symptoms in poisoning in a murine model [11]. As TsV induces an exacerbated inflammatory response in different organs, and inflammation is an important facet during scorpionism, it was hypothesized that the immune response also played a role in pancreatic dysfunction, since this organ is exposed to high concentrations of venom in the first 24 h after the accident [12].

Data reported in this study demonstrate that a decompensation in insulin levels does not occur in *Il1r1*^−/−^ mice. Moreover, the absence of hyperglycemia is correlated with diminished NO levels in the pancreas of envenomed *Il1r1*^−/−^ mice. This suggests that IL1/IL-1R signaling induces NO production in the pancreas after scorpion envenomation. Moreover, IL-1β induces NO production and disrupts insulin production by β-cells [21,22]. In fact, NO impacts mitochondrial oxidative phosphorylation, which, in turn, inhibits insulin secretion [21]. Mild edema was also observed in β-cells of islets with notable leukocyte infiltration, which has been shown to reduce insulin secretion in different mouse models [23,24,25,26]. This study speculated that the inflammatory infiltrate in the pancreas contributes to increased IL-1 production, whereas IL-1/IL-1R signaling disrupts β-cell homeostasis via the induction of NO.

In summary, our study shows that TsV induces IL-1α and IL-1β production in the pancreas. These cytokines signal via IL-1R, which induces NO production and disrupts insulin secretion during severe scorpion envenomation. To our knowledge, this is the first study to address the mechanism by which scorpion envenomation induces hyperglycemia. Importantly, as elevated glucose levels can influence the development of severe manifestations, this study provides novel insights into the design of supportive therapy with NO inhibitors combined with antiscorpion venom serum to overcome fatal outcomes of scorpion envenomation. 

## 4. Materials and Methods

### 4.1. TsV

*Ts* (yellow scorpion) venom was obtained from Butantan Institute, São Paulo, SP, Brazil. The venom was extracted by electric stimulation (12 mV), followed by freeze-drying; the venom was then stored at –20 °C. For animal inoculation, TsV was weighed and diluted in sterile PBS (1 mg/mL), and filtered through 0.22-µm sterilizing membrane (Millipore, USA). Before use, TsV was tested for endotoxin contamination using limulus amebocyte lysate (LAL) test (QCL-1000, BioWhittaker, Cambrex Corporation, East Rutherford, NJ, USA). Endotoxin was absent in all the TsV samples.

### 4.2. Animals

For in vivo studies, C57BL/6 and *Il1r1*^−/−^ adult mice (6–8 weeks), obtained from the animal facilities of the Faculdade de Medicina de Ribeirão Preto, University of São Paulo, Brazil, were maintained under a light/dark cycle with free access to food and water. Prior to the experiments, the animals were fasted for 8 h. Maintenance of the mice and the experiments were conducted in accordance with the Ethical Principles in Animal Research adopted by the National Council for the Control of Animal Experimentation and approved by the Animal Care and Use Committee of the Faculdade de Ciências Farmacêuticas de Ribeirão Preto, at the Universidade de São Paulo, Ribeirão Preto, São Paulo, Brazil (Process No.: 16.1.1081.60.5, approved on 4 September 2017).

### 4.3. In Vivo Experiments and Drug Treatments

Mice were inoculated with a lethal dose of TsV, i.e., 180 µg.kg^−1^ intraperitoneally (i.p.), as described in a previous study [11]. The humane endpoint used in this study was 60 min after TsV inoculation. Following this, the mice were anesthetized with ketamine hydrochloride (75 mg.kg^−1^) and xylazine hydrochloride (10 mg.kg^−1^), and then euthanized by cervical displacement. Mice inoculated with PBS (300 µL/i.p.) were used as negative control. Pancreas and blood were isolated for histological analysis and quantification of inflammatory mediators. For L-NAME treatment, animals were treated 15 min before TsV or PBS inoculation with a 100-mg.kg^−1^ i.p. dose.

### 4.4. Glucose Measurement

For glucose measurement, PBS-inoculated animals and mice challenged with a lethal dose of venom were anesthetized after 60 min of PBS or TsV inoculation, and a tail puncture was performed for blood extraction. Next, blood droplets were placed on blood glucose test strips coupled to a FreeStyle Optium Neo meter (Abbott Diabetes Care Inc., Witney, United Kingdom), and the results observed were expressed in milligram per deciliter. All animals were fasted 8 h prior to PBS or TsV inoculation and glucose measurement.

### 4.5. Quantification of Insulin, Cytokines, MPO, and NO

Serum obtained from mice challenged with venom lethal dose or PBS-inoculated animals was used for insulin measurement by enzyme-linked immunosorbent assay (ELISA), according to the manufacturer’s instructions (Insulin Mouse ELISA Kit, Thermo Fisher, Waltham, MA, USA). Cytokines/chemokines (IL-1α, IL-1β, CCL2, and CCL3) and MPO were quantified in pancreas homogenates or serum by ELISA, according to the manufacturer’s instructions (DuoSet ELISA Kit, R&D Systems, Minneapolis, MN, USA). The amount of nitrite present in the supernatants was measured as an indicator of NO production using the Griess method, and a standard curve was plotted using serial NaNO_2_ dilutions. The assay was performed in quadruplicate, and the absorbance at 540 nm was recorded 10 min after addition of NaNO_2_.

### 4.6. Immunohistochemistry

Pancreas of lethally dosed and PBS-inoculated mice was removed after 1 h of envenomation, fixed in 10% buffer formalin, and then imbedded in paraffin. Sections of 5 µm were dewaxed and incubated with peroxidase-blocking reagent. The slides were treated with 1% of bovine serum albumin (BSA) for blocking unspecific binding. Anti-insulin antibody clone [EPR17359], RabMab (Abcam, Burlingame, CA, USA), was applied to the sections followed by enhanced luminol-based (ECL) antirabbit-horseadish peroxidase (HRP) (GE Healthcare, Chicago, IL, USA). 

### 4.7. Statistical Analysis

All results are expressed as mean ± standard deviation. Data were analyzed using one-way analysis of variance followed by Bonferroni posttest. All analyses were performed using GraphPad Prism software (GraphPad, San Diego, CA, United States). Differences were considered statistically significant when *p* < 0.05.
